# Impact of rhinitis on asthma severity in school-age children

**DOI:** 10.1111/all.12467

**Published:** 2014-08-04

**Authors:** M Deliu, D Belgrave, A Simpson, C S Murray, G Kerry, A Custovic

**Affiliations:** 1 Centre for Respiratory Medicine and Allergy, Institute of Inflammation and Repair, University of Manchester & University Hospital of South ManchesterManchester, UK; 2 Centre for Health Informatics, Institute of Population Health, University of ManchesterManchester, UK

**Keywords:** asthma severity, childhood asthma, childhood rhinitis

## Abstract

**Background:**

In a population-based sample of school-age children, we investigated factors associated with rhinitis, and differences between allergic and nonallergic rhinitis. Amongst children with asthma, we explored the association between rhinitis and asthma severity.

**Methods:**

Children participating in a birth cohort study (*n* = 906) were reviewed at age 8 years. Asthma was defined as at least two of the following three features: physician-diagnosed asthma, currently using asthma medication and current wheeze. We measured lung function (plethysmography and spirometry) and airway hyper-reactivity (AHR; methacholine challenge).

**Results:**

In the analysis adjusted for the presence of asthma, children with rhinitis had significantly higher AHR (*P* = 0.001). Maternal smoking and absence of breastfeeding were stronger predictors of nonallergic rhinitis, whereas current wheeze and eczema were stronger predictors of allergic rhinitis. Amongst asthmatics (*n* = 159), when compared to 76 children without rhinitis, those with rhinitis (*n* = 83) were 2.89-fold (95% CI 1.41–5.91) more likely to experience frequent attacks of wheezing, 3.44-fold (1.19–9.94) more likely to experience severe attacks of wheezing limiting speech, 10.14-fold (1.27–81.21) more likely to have frequent visits to their doctor because of asthma and nine-fold (1.11–72.83) more likely to miss school. Reported use of intranasal corticosteroids resulted in a numerically small, but consistent reduction in risk, rendering the associations between rhinitis and asthma severity nonsignificant.

**Conclusion:**

We observed differences in risk factors and severity between allergic and nonallergic rhinitis. In children with asthma, rhinitis had adverse impact on asthma severity. The use of intranasal corticosteroids resulted in a small, but consistent reduction in the risk.

Although rhinitis is one of the most common chronic diseases in childhood 1,2, only a minority of symptomatic children have appropriate diagnosis 3,4. The burden of the disease to individual patients and the society is often underestimated, and there is a general paucity of data on the risk factors and phenotypes of rhinitis in childhood 1. Numerous epidemiological studies have demonstrated a link between rhinitis and asthma 1, with rhinitis often preceding asthma development 5. There is evidence to suggest that amongst adult asthmatics, those with comorbid rhinosinusitis have poorer quality of life 6, and chronic rhinitis may be an important comorbidity of severe asthma 7. A cross-sectional study amongst children and adolescents with moderate/severe asthma treated with inhaled corticosteroids (ICS) suggested that concurrent allergic rhinitis may be a risk factor for the use of emergency care services 8. A recent cross-sectional survey amongst 203 children with asthma has reported that allergic rhinitis in this patient group is common and has an adverse impact on asthma control 9. However, most previous studies on the impact of rhinitis on childhood asthma severity have been carried out in selected populations of asthmatics attending secondary/tertiary care specialist services 8,9, and the effect at a population level is unknown.

We aimed to investigate the risk factors for rhinitis in a population-based sample of school-age children participating in an unselected birth cohort and whether there are differences in risk factors between allergic and nonallergic rhinitis. Amongst children with asthma, we investigated the association between rhinitis and asthma severity.

## Methods

### Study design

Manchester Asthma and Allergy Study is a population-based birth cohort 10. Subjects were recruited antenatally and followed prospectively. The study was approved by the local research ethics committee. Parents/guardians gave written informed consent.

### Data sources

#### Clinical follow-up

Participants attended follow-up at age 8 years. Validated ISAAC questionnaire 11 was interviewer-administered to collect information on parentally reported symptoms, doctor diagnoses and environmental exposures. We performed skin prick tests to common inhalant allergens (mite, cat, dog, grass and tree pollen [Bayer, Elkhart, USA]).

#### Medical records data

We extracted all data from electronic and paper-based primary care medical records, including prescriptions of ICS and intranasal corticosteroids (INCS) between January 2006 and February 2008 (median age 10 years).

#### Lung function, airway hyper-reactivity and airway inflammation

We measured specific airway resistance (sR_aw_) using plethysmography 12. We used spirometry to measure FEV_1_ (expressed as % predicted) and FVC 13. Airway hyper-reactivity (AHR) was assessed using methacholine challenge (five-step protocol with quadrupling doses of methacholine) 14. We calculated methacholine dose–response slope (MDRS) to include all evaluable data as a continuous variable 15. Fractional exhaled nitric oxide (FeNO) was measured as an indicator of airway inflammation 16.

### Definition of variables

#### Atopy

Mean wheal diameter ≥3 mm than the negative control to at least one allergen.

#### Current rhinitis

Positive response to the question ‘In the past 12 months, has your child ever had a problem with sneezing, or a runny nose, or a blocked nose when he/she did not have a cold or the flu?' We designated current rhinitis in the presence of atopy as *allergic rhinitis*, whilst *nonallergic rhinitis* was defined as current rhinitis in the absence of atopy.

#### Current wheeze

Positive response to the question ‘Has your child had wheezing or whistling in the chest in the last 12 months?’

#### Current asthma

At least two of the following three features: (i) current wheeze, (ii) current use of asthma medication and (iii) physician-diagnosed asthma ever.

#### Wheeze phenotypes

Using prospectively collected data, we defined wheeze phenotypes as no wheezing, transient-early wheezing, intermittent wheezing, late-onset wheezing and persistent wheezing 17.

#### Current maternal smoking

Positive response to the question ‘Does the child's mother smoke at present?’

### Markers of asthma severity

#### Number of wheeze attacks

Parentally reported number of attacks of wheezing their child had in the past 12 months. Troublesome asthma was defined as four or more attacks.

#### Nocturnal awakenings

Ascertained by the question ‘In the past 12 months, how often, on average has your child's sleep been disturbed due to wheezing?’ We defined troublesome asthma as one or more nights with disturbed sleep per week.

#### Wheeze limiting speech

Positive answer to the question ‘In the past 12 months, has wheezing been severe enough to limit your child's speech to only one or two words at a time between breaths?’

#### Visits to family doctor for wheeze/asthma

Troublesome asthma was defined as four or more visits in the past 12 months.

#### School days missed due to wheezing/asthma

Parentally reported number of school days the child missed in the past 12 months. We defined troublesome asthma as 6 or more days.

### Statistical analysis

Univariate and multivariate analyses were carried out using logistic regression (SPSS 20.0, Chicago, IN, USA). Analysis was carried out assuming that covariate data were missing completely at random. The effect size was measured using odds ratios (OR) and 95% confidence interval (CI). All models were adjusted for gender.

## Results

### Participant flow and demographic data

**Figure 1:**
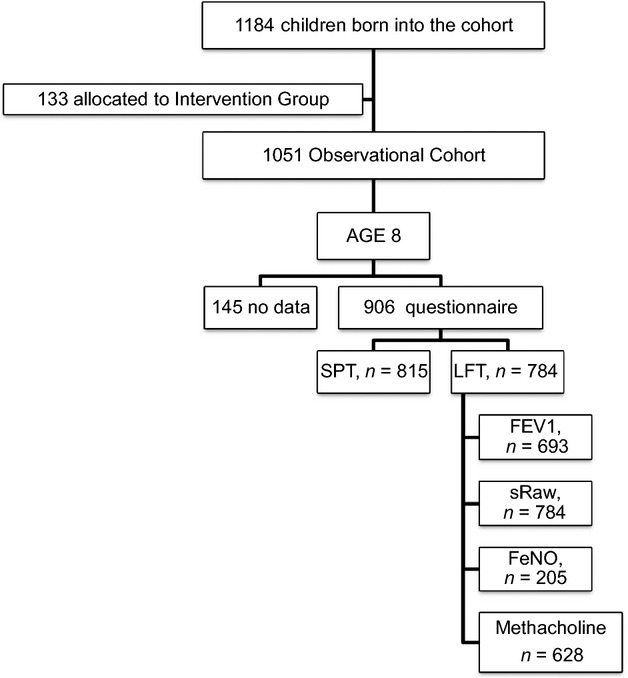
Participant flow.

Of 1184 children born into the study, 133 were allocated to an intervention group and excluded from this analysis 18. Questionnaire data were available for 906 children, of whom 815 underwent skin testing, 724 lung function testing, 628 methacholine challenges and 205 FeNO measurements (Fig.1). Current rhinitis was present in 260 of 906 (29%) children, of whom 229 had skin tests data (140 were designated as having allergic, and 89 as nonallergic rhinitis). Of the 905 (18%) participants, 159 had asthma; 259 of 815 (31.7%) were atopic. Figure S1 shows the prevalence and overlap of comorbid current rhinitis, asthma and eczema.

### Factors associated with rhinitis

Results of the univariate analysis are shown in Table S1. We performed multiple logistic regression analysis including all factors significantly associated with current rhinitis in the univariate analysis. Wheeze in the first year of life (OR 1.53, 95% CI 1.04–2.24, *P* = 0.03), current eczema (1.90, 1.24–2.91, *P* = 0.003), and sensitization to grass (3.87, 2.26–6.64, *P* < 0.001) and cat (2.05, 1.09–3.86, *P* = 0.03) remained independent associates of rhinitis. We then ascertained the differences in risk factors between allergic and nonallergic rhinitis (Table1). Maternal smoking and having never been breastfed were significantly stronger predictors of nonallergic rhinitis, whereas current wheeze and eczema were significantly stronger predictors of allergic rhinitis. Using interference with daily activities as a marker of severity, allergic rhinitis appeared to be more severe than nonallergic rhinitis (Table1). As expected, allergic rhinitis was more likely to have a seasonal pattern.

**Table 1:** Associates of allergic and nonallergic rhinitis

	Allergic rhinitis *n* (%)	Nonallergic rhinitis *n* (%)	*P*-value
Never breastfed	30/130 (23.1)	32/88 (36.4)	0.03
Current maternal smoking	18/140 (12.9)	23/89 (25.8)	0.01
Wheeze ever	90/140 (64.3)	44/89 (49.4)	0.02
Current wheeze (age 8 years)	68/140 (48.6)	12/89 (13.5)	<0.001
Persistent wheeze	46/140 (32.9)	14/89 (15.7)	0.009
Current eczema (age 8 years)	70/140 (50.0)	19/86 (22.1)	<0.001
Eczema in the first year	74/125 (59.2)	28/87 (32.2)	<0.001
Interference with daily activities	79/140 (56.4)	35/89 (39.3)	0.01
Spring/summer pattern	90/140 (64.3)	41/89 (46.1)	0.009
Perennial	27/140 (19.3)	19/89 (21.3)	0.20

Both wheeze and asthma were strongly associated with rhinitis, but different associations were observed for different wheeze phenotypes. We observed strong associations with persistent and late-onset wheeze, but there was no association between rhinitis and transient-early wheezing; these associations appeared stronger for allergic than nonallergic rhinitis (Table S2).

In the analysis adjusted for the presence of asthma, children with rhinitis had significantly higher AHR (MDRS; mean difference [95% CI], −0.85 [−1.34 – (−)0.36], *P* = 0.001) and FeNO (mean difference [95% CI], 0.37 [0.21–0.53], *P* < 0.001); these associations were significantly stronger for allergic than nonallergic rhinitis (Table S3). Amongst atopic children, there was no difference in FeNO between those with and without rhinitis (mean difference [95% CI], 0.23 [−0.09 to 0.57], *P* = 0.17). We found no difference in lung function (sRaw, FEV_1_ and FEV_1_/FVC) between children with and without rhinitis.

### Rhinitis and asthma severity

Amongst 159 children with asthma, 83 had contemporaneous rhinitis. There was no significant difference in AHR between children with rhinitis compared to those without; similarly, we observed no difference in lung function (Table S4). An apparent difference in FeNO between asthmatic children with and without rhinitis was no longer significant after adjustment for atopy (*P* = 0.16).

**Figure 2:**
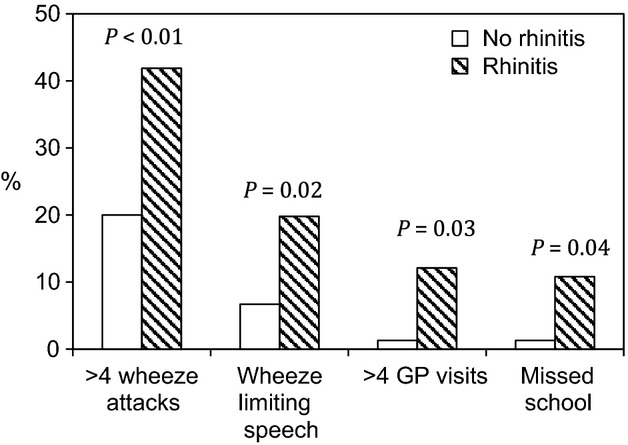
Rhinitis and markers of asthma severity amongst 159 children with asthma.

In contrast, we observed marked differences between asthmatic children with and without rhinitis in the frequency of wheeze attacks, wheeze limiting speech, unscheduled visits to family doctor because of poor asthma control, and missed school days (Fig.2). Compared to asthmatic children without rhinitis, those with both asthma and rhinitis were:

- 2.89-fold (95% CI 1.41–5.91) more likely to experience frequent attacks of wheezing (*P* < 0.01).
- 3.44-fold (95% CI 1.19–9.94) more likely to experience severe attacks of wheezing limiting speech (*P* = 0.02).
- 10.14-fold (95% CI 1.27–81.21) more likely to have frequent unscheduled visits to their doctor because of asthma (*P* = 0.03).
- 9-fold (95% CI 1.11–72.83) more likely to miss school because of their asthma (*P* = 0.04).

A trend that failed to reach statistical significance was observed for nocturnal awakenings (OR 1.81, 95% CI 0.79–4.12, *P* = 0.16).

### Influence of treatment for rhinitis on the association of rhinitis with asthma severity

We carried out an exploratory analysis to ascertain whether treatment for rhinitis modifies the association of rhinitis with asthma severity, using multivariate analyses adjusted for the use of rhinitis medication (Table2). Adjusting the analysis for parentally reported use of antihistamines did not remove the impact of rhinitis or modify the magnitude of the risk associated with any of the markers of asthma severity. In contrast, reported use of intranasal corticosteroids (INCS) resulted in a numerically small, but consistent reduction in the risk of frequent wheeze attacks, wheeze limiting speech, frequent GP visits and school days missed, rendering these associations between rhinitis and asthma severity nonsignificant. We observed similar reductions in risks after adjusting the analysis for the use of ICS (Table2), whilst adjustment for both INCS and ICS appeared to further minimize the impact of rhinitis on asthma severity (Table S5).

**Table 2:** Rhinitis and markers of asthma severity after medication adjustment

	No rhinitis *n* (%)	Current rhinitis *n* (%)	OR	95% CI	*P*-value
≥4 wheezing attacks	15/75 (20.0)	34/81 (41.9)	2.89	1.41–5.91	<0.01
Adjusted for antihistamines			2.93	1.42–6.03	<0.01
Adjusted for INCS			2.23	0.78–6.38	0.13
Adjusted for ICS			1.61	0.59–4.37	0.35
Wheeze limiting speech	5/75 (6.7)	16/81 (19.8)	3.44	1.19–9.94	0.02
Adjusted for antihistamines			3.62	1.25–10.51	0.02
Adjusted for INCS			3.19	0.79–12.89	0.10
Adjusted for ICS			2.13	0.64–7.05	0.22
≥4 visits to GP for asthma	1/75 (1.3)	10/83 (12.1)	10.14	1.27–81.21	0.03
Adjusted for antihistamines			10.35	1.28–83.34	0.03
Adjusted for INCS			7.13	0.83–61.17	0.07
Adjusted for ICS			7.68	0.89–6.17	0.06
≥6 school days missed	1/75 (1.3)	9/83 (10.8)	9.00	1.11–72.83	0.04
Adjusted for antihistamines			8.98	1.23–81.00	0.03
Adjusted for INCS			8.16	0.96–69.21	0.054
Adjusted for ICS			8.03	0.93–9.67	0.06

## Discussion

### Key findings

Our population-based study demonstrated that amongst children with asthma, the presence of rhinitis has significant adverse effect on asthma severity. Amongst asthmatic children, contemporaneous rhinitis was associated with more frequent wheeze attacks (2.4-fold increase in risk), more severe attacks of wheezing associated with speech limitations (3.4-fold increase in risk), more frequent visits to the family doctor (9.5-fold increase in risk) and greater school absenteeism because of asthma (nine-fold increase in risk). Whereas adjusting for the use of antihistamines did not change these associations, adjusting for the use of INCS resulted in small, but consistent reductions in risk, rendering the associations between rhinitis and markers of asthma severity nonsignificant.

We observed differences in the associated risk and severity between allergic and nonallergic rhinitis. Having never been breastfed and being exposed to maternal smoking were stronger associates of nonallergic than allergic rhinitis, whereas current wheeze and eczema were significantly stronger predictors of allergic rhinitis, suggesting different causal pathways.

### Limitations

We relied on parental reporting of child's symptoms and acknowledge potential limitations of this approach 19. We collected data on medication prescribed by primary care physicians; this, however, does not confirm that the treatments were taken by children. We were unable to obtain data on all outcomes (including answers to all questions, skin tests, lung function, AHR and FeNO) for all children; however, we found no significant differences in demographic characteristics between children with and without these data, suggesting that it is unlikely that this has influenced the results (not presented, available on request). We acknowledge that our definition of allergic rhinitis is an epidemiological one and that demonstration of the cause/effect relationship between exposure to sensitizing allergen and symptom would be preferable; however, such investigations are beyond the remit of the present study. Finally, although we carried out the analyses amongst children participating in a longitudinal birth cohort study, due to cross-sectional nature of these data, the results should be interpreted with caution.

One strength of our study is the large sample of children, representative of and generalizable to the general population, with an excellent retention and high follow-up rate.

### Interpretation

#### Prevalence, risk factors and characteristics

Our results confirm that rhinitis is common in school-age children and that it frequently coexists with asthma 20,21. There was a strong association between rhinitis with current and persistent, but not transient-early wheezing, suggesting that different wheezing phenotypes may arise through different mechanisms 19,22. The results appear intuitively correct, with maternal smoking and the absence of breastfeeding being stronger associates of nonallergic, and wheeze and eczema of allergic rhinitis. These data suggest that there may be different phenotypes of rhinitis during childhood, with both shared and unique developmental pathways 23. Longitudinal studies may help identification of such phenotypes.

We found that children with allergic rhinitis had more severe symptoms than those with nonallergic rhinitis. Similarly, a significant (but underestimated) burden of allergic rhinitis has been reported previously in a large-scale survey in the USA 24 and amongst children with asthma from Turkey 25.

Allergic rhinitis has been shown to increase FeNO, suggesting increased lower airway inflammation 26,27. In contrast to these studies, amongst atopic children in our study, there was no association between FeNO and rhinitis. We observed no differences in lung function between children with and without rhinitis. However, in agreement with the previous study by Koh et al., 28 we demonstrated higher airway reactivity amongst children with rhinitis, even after adjusting for the presence of asthma. Similarly, data from cross-sectional 29 and population-based studies 30,31 in adults have suggested a strong association between allergic rhinitis and lower airway dysfunction.

#### Rhinitis and asthma severity

In a recent important cross-sectional study amongst children with asthma recruited from the hospital asthma clinic, the presence of allergic rhinitis had significant adverse effect on asthma control, even when asthma was considered adequately controlled 9. We have extended these observations to the unselected population of children with asthma and to a number of other important markers of asthma severity (including frequency and severity of wheeze attacks, unscheduled use of medical care and school absenteeism). Of particular note is our finding that children with asthma and rhinitis have ninefold increase in the risk of missing school compared to children with asthma only.

In both ours and the study by de Groot et al. 9, treatment with INCS appeared to modify the association between rhinitis and asthma severity. The observed reduction in risk was not due to missing observations. This is consistent with findings in a retrospective cohort of older children and adults, which showed that patients who were treated for allergic rhinitis were significantly less likely to visit emergency departments or be hospitalized than those who were not treated 32. However, this study did not differentiate between antihistamines and INCS. One could argue that children with asthma and rhinitis who receive appropriate rhinitis treatment with INCS may also be prescribed better asthma treatment, such as ICS. However, the apparent effect of INCS on asthma severity in our study remained mostly unchanged when we adjusted the analysis for the use of ICS, suggesting that it is unlikely that the observed reduction in asthma severity amongst children using INCS is confounded by better treatment for asthma. These findings suggests (but do not prove) that amongst children with asthma and rhinitis, appropriate treatment of rhinitis with INCS may improve asthma control. The definitive answer can only be obtained in appropriately designed randomized controlled trials; however, there are as yet no such long-term trials in children 33. A recent 4-week study amongst children with mild/moderate asthma and intermittent allergic rhinitis has shown that INCS may improve exercise-induced bronchospasm 34. In contrast, a double-blind randomized crossover trial amongst adults with asthma and persistent allergic rhinitis did not demonstrate any steroid-sparing effect of adding INCS to low-dose ICS on lower airway outcomes 35. Recent meta-analysis of 18 studies assessing the effect of INCS on asthma outcomes in patients with AR and comorbid asthma concluded that INCS may improve some lower airway outcomes, but that further studies are needed to confirm the role of INCS sprays as therapy for asthma outcomes 33.

In conclusion, we observed differences in risk factors and severity between allergic and nonallergic rhinitis amongst school-age children. In children with asthma, the presence of rhinitis had adverse impact on asthma severity. Adjustment for the use of INCS resulted in a small, but consistent reduction in the risk.

## Supporting Information

Figure S1 (A) Prevalence of current rhinitis, asthma, and eczema in the study population; and (B) Proportional Venn diagram showing the overlap of co-morbid current rhinitis, asthma and eczema. **Table S1.** Risk factors for rhinitis (univariate analysis). **Table S2.** Association of rhinitis with asthma, current wheeze and childhood wheeze phenotypes. **Table S3.** Lung function (sRaw, FEV_1_ and FEV_1_/FVC), airway hyper-reactivity (methacholine DRS-MDRS) and FeNO at age 8 years in children with and without rhinitis (analysis adjusted for asthma). **Table S4.** Lung function (sRaw, FEV_1_ and FEV_1_/FVC), airway hyper-reactivity (methacholine DRS) and FeNO at age 8 years amongst asthmatic children with and without rhinitis. **Table S5.** Rhinitis and markers of asthma severity, among asthmatic children, after adjustment for the use of inhaled (ICS) and intranasal (INCS) corticosteroid.
