# Supplementary material for: Impact of rhinitis on asthma severity in school-age children
Source: Allergy. 2014 Aug 4;69(11):1515–21. doi: 10.1111/all.12467 (PMC4209798; doi:10.1111/all.12467)
Supplement: Figure S1 — (A) Prevalence of current rhinitis, asthma, and eczema in the study population; and (B) Proportional Venn diagram showing the overlap of co-morbid current rhinitis, asthma and eczema. Table S1. Risk factors for rhinitis (univariate analysis). Table S2. Association of rhinitis with asthma, current wheeze and childhood wheeze phenotypes. Table S3. Lung function (sRaw, FEV1 and FEV1/FVC), airway hyper-reactivity (methacholine DRS-MDRS) and FeNO at age 8 years in children with and without rhinitis (analysis adjusted for asthma). Table S4. Lung function (sRaw, FEV1 and FEV1/FVC), airway hyper-reactivity (methacholine DRS) and FeNO at age 8 years amongst asthmatic children with and without rhinitis. Table S5. Rhinitis and markers of asthma severity, among asthmatic children, after adjustment for the use of inhaled (ICS) and intranasal (INCS) corticosteroid. [file all0069-1515-sd1.docx]

**IMPACT OF RHINITIS ON ASTHMA SEVERITY IN SCHOOL-AGE CHILDREN**

Matea Deliu^1^, Danielle Belgrave^1, 2^, Angela Simpson^1^, Clare S Murray^1^, Gina Kerry^1^, Adnan Custovic^1^

^1^Centre for Respiratory Medicine and Allergy, Institute of Inflammation and Repair, University of Manchester & University Hospital of South Manchester, Manchester, UK

^2^Centre for Health Informatics, Institute of Population Health, University of Manchester, UK

**ONLINE DATA SUPPLEMENT**

**Table E1**. Risk factors for rhinitis (univariate analysis);

|  | No Rhinitis  n (%) | Current Rhinitis  n (%) | OR | 95% CI | p-value |
| --- | --- | --- | --- | --- | --- |
| **Parental atopy** | 491/631 (77.8) | 221/245 (90.2) | 2.55 | 1.61-4.05 | **<0.001** |
| **Maternal hay fever** | 136/646 (21.1) | 87/260 (33.5) | 1.88 | 1.37-2.59 | **<0.001** |
| **Paternal hay fever** | 126/646 (19.5) | 72/260 (27.7) | 1.57 | 1.13-2.20 | **0.008** |
| **Maternal asthma ever** | 108/646 (16.7) | 58/260 (22.3) | 1.43 | 1.00-2.05 | **0.05** |
| **Maternal eczema** | 92/646 (14.2) | 51/260 (19.6) | 1.47 | 1.01-2.14 | **0.05** |
| **Current eczema (age 8)** | 121/643 (18.8) | 98/257 (38.1) | 2.66 | 1.93-3.66 | **<0.001** |
| **Eczema in the first year of life** | 189/610 (31.0) | 113/239 (47.3) | 1.99 | 1.47-2.71 | **<0.001** |
| **Wheeze in the first year of life** | 196/633 (31.0) | 107/253 (42.3) | 1.63 | 1.21-2.21 | **0.001** |
| **Sensitization to grass (age 8)** | 45/586 (7.7) | 83/229 (36.2) | 6.84 | 4.55-10.26 | **<0.001** |
| **Sensitization to cat (age 8)** | 34/586 (5.8) | 54/229 (23.6) | 5.01 | 3.16-7.95 | **<0.001** |

**Table E2**. Association of rhinitis with asthma, current wheeze and childhood wheeze phenotypes.

|  | No rhinitis  n (%) | Current rhinitis  n (%) | OR | 95% CI | p-value | Allergic rhinitis n (%) | Non-allergic rhinitis; n (%) | OR | 95% CI | p-value |
| --- | --- | --- | --- | --- | --- | --- | --- | --- | --- | --- |
| **Asthma ever** | 123/646 (19.0) | 97/260 (37.3) | 2.59 | 1.88-3.57 | **<0.001** | 56/140 (40) | 30/89 (33.7) | 1.34 | 0.77-2.34 | **0.29** |
| **Current wheeze (age 8)** | 75/645 (11.6) | 87/260 (33.5) | 3.83 | 2.69-5.45 | **<0.001** | 68/140 (48.6) | 12/89 (13.5) | 0.17 | 0.08-0.33 | **<0.001** |
| ***Wheeze phenotypes*** |  |  |  |  |  |  |  |  |  |  |
| **Transient wheeze** | 68/643 (10.6) | 35/259 (13.5) | 1.23 | 0.83-1.83 | 0.29 | 21/140 (15) | 24/89 (26.9) | 0.73 | 0.34-1.56 | 0.42 |
| **Intermittent wheeze** | 68/643 (10.6) | 35/259 (13.5) | 2.06 | 1.27-3.32 | **0.003** | 16/140 (11.4) | 14/89 (15.7) | 0.96 | 0.41-2.27 | 0.92 |
| **Late onset wheeze** | 25/643 (3.9) | 26/259 (10.0) | 4.16 | 2.28-7.59 | **<0.001** | 20/140 (14.3) | 6/89 (6.7) | 2.79 | 0.99-7.82 | 0.051 |
| **Persistent wheeze** | 59/643 (9.2) | 65/259 (25.1) | 4.41 | 2.87-6.78 | **<0.001** | 46/140 (32.9) | 14/89 (15.7) | 2.75 | 1.28-5.92 | **0.009** |

**Table E3:** Lung function (sRaw, FEV_1_ and FEV_1_/FVC), airway hyper-reactivity (methacholine DRS-MDRS) and FeNO at age 8 years in children with and without rhinitis (analysis adjusted for asthma)

|  | No rhinitis  Number, GM (95% CI) | Current rhinitis  Number , GM (95% CI) | p-value | Allergic rhinitis  Number , GM (95% CI) | Non-allergic rhinitis  Number , GM (95% CI) | p-value |
| --- | --- | --- | --- | --- | --- | --- |
| sRaw (kPa·s^-1^) | 561, 1.22 (1.20 – 1.24) | 233, 1.21 (1.18 – 1.24) | 0.11 | 125, 1.21 (1.17-1.25) | 85, 1.21 (1.16-1.26) | 0.37 |
| FEV_1_ (% pred) | 496, 98.26 (97.18 – 99.35) | 197, 98.38 (96.71 – 100.08) | 0.46 | 117, 97.34 (95.01-99.72) | 73, 99.77 (97.34-102.26) | 0.38 |
| FEV_1_/FVC | 497, 86.18 (85.64-86.73) | 196, 86.18 (83.33-87.04) | 0.32 | 116, 85.50 (84.43-86.59) | 73, 87.31 (85.81-88.84) | 0.18 |
| MDRS | 388, 4.82 (4.44 – 5.25) | 165, 3.58 (3.06 – 4.18) | **0.001** | 95, 3.11 (2.52-3.83) | 65, 4.33 (3.38-5.53) | **0.006** |
| FeNO (ppb) | 279, 9.39 (8.76 – 10.07) | 97, 14.29 (12.18 – 16.94) | **<0.001** | 53, 23.58 (18.77 – 29.61) | 41, 10.21 (8.19 – 12.71) | **0.01** |

**Table E4:** Lung function (sRaw, FEV_1_ and FEV_1_/FVC), airway hyper-reactivity (methacholine DRS) and FeNO at age 8 years amongst asthmatic children with and without rhinitis.

|  | No Rhinitis  Number, GM (95% CI) | Current Rhinitis  Number, GM (95% CI) | p-value | Allergic rhinitis  Number, GM (95% CI) | Non-allergic rhinitis  Number, GM (95% CI) | p-value |
| --- | --- | --- | --- | --- | --- | --- |
| sRaw (kPa·s^-1^) | 69, 1.33 (1.26 – 1.41) | 68, 1.27 (1.20 – 1.34) | 0.21 | 54, 1.27 (1.20-1.35) | 13,1.28 (1.12-1.48) | 0.90 |
| FEV_1_ (% pred) | 61, 94.25 (90.28 – 98.41) | 64, 96.79 (93.29 – 100.42) | 0.36 | 55, 96.44 (92.46-100.59) | 8, 99.79 (93.14-106.91) | 0.64 |
| FEV_1_/FVC | 62, 82.93 (80.84-85.07) | 63, 84.85 (83.26-86.47) | 0.18 | 54, 84.53 (82.81-86.28) | 8, 86.34 (80.89-92.15) | 0.45 |
| MDRS | 53, 2.90 (2.01 – 4.19) | 49, 2.95 (2.18 – 3.99) | 0.69 | 42, 2.86 (2.02-4.05) | 6, 3.23 (1.63-6.43) | 0.43 |
| FeNO (ppb) | 33, 15.33 (11.13 – 21.98) | 23, 26.31 (17.11 – 42.52) | 0.05 | 17, 37.52 (26.05-54.05) | 6, 16.98 (3.92-73.63) | 0.64 |

**Table E5.** Rhinitis and markers of asthma severity, among asthmatic children, after adjustment for the use of inhaled (ICS) and intranasal (INCS) corticosteroid

|  | No rhinitis  n (%) | Current rhinitis  n (%) | OR | 95% CI | p-value |
| --- | --- | --- | --- | --- | --- |
| **≥4 wheezing attacks** | 15/75 (20.0) | 34/81 (41.9) | 2.89 | 1.41-5.91 | **<0.01** |
| Adjusted for INCS & ICS |  |  | 2.17 | 0.75-6.31 | 0.16 |
| **Wheeze limiting speech** | 5/75 (6.7) | 16/81 (19.8) | 3.44 | 1.19-9.94 | **0.02** |
| Adjusted for INCS & ICS |  |  | 1.98 | 0.49-7.88 | 0.33 |
| **≥4 visits to GP for asthma** | 1/75 (1.3) | 10/83 (12.1) | 10.14 | 1.27-81.21 | **0.03** |
| Adjusted for INCS & ICS |  |  | N/A |  |  |
| **≥6 school days missed** | 1/75 (1.3) | 9/83 (10.8) | 9.00 | 1.11-72.83 | **0.04** |
| Adjusted for INCS & ICS |  |  | 3.71 | 0.36-38.37 | 0.27 |

**Figure E1**. A) Prevalence of current rhinitis, asthma, and eczema in the study population; and B) Proportional Venn diagram showing the overlap of co-morbid current rhinitis, asthma and eczema.

A)


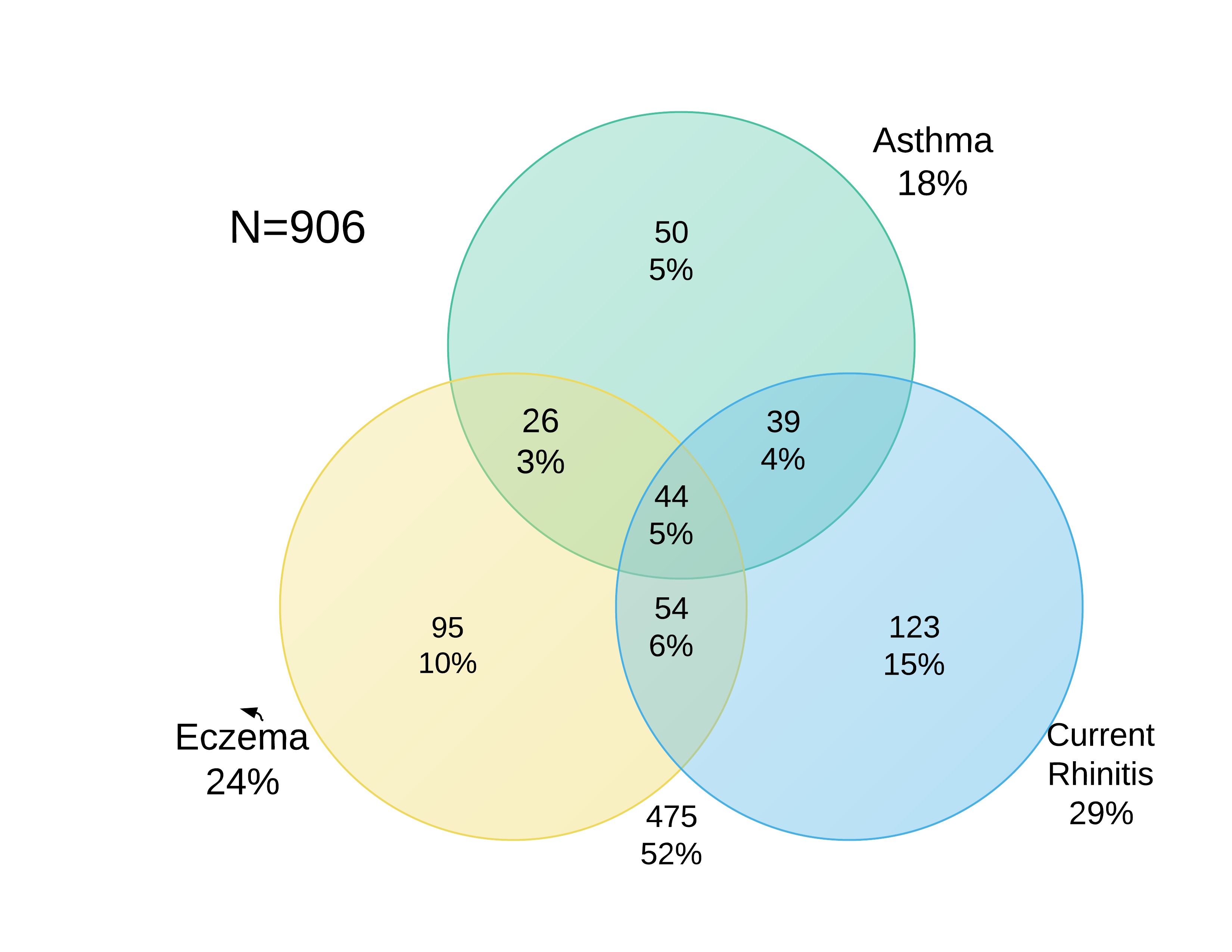


B)

**
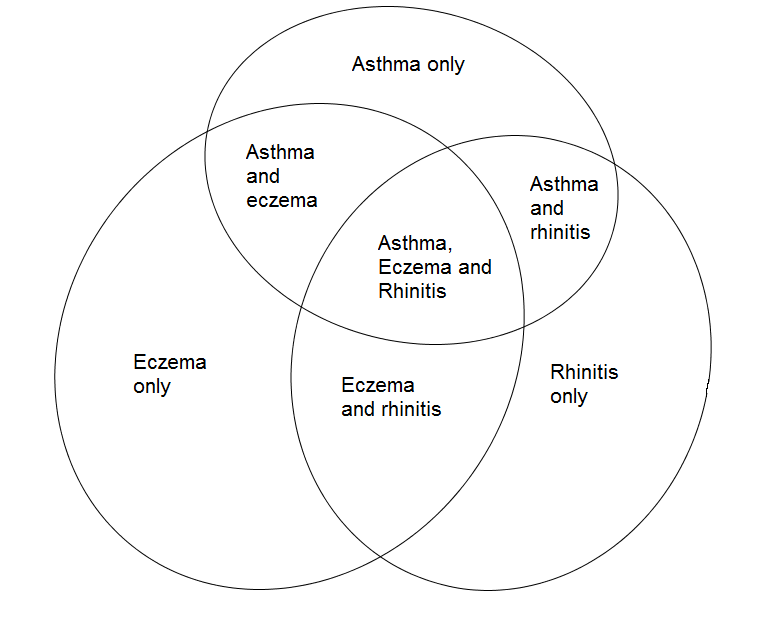
**
